# Identification of subpopulations in mesenchymal stem cell-like cultures from human umbilical cord

**DOI:** 10.1186/1478-811X-7-6

**Published:** 2009-03-20

**Authors:** Ingrida Majore, Pierre Moretti, Ralf Hass, Cornelia Kasper

**Affiliations:** 1 Institute of Technical Chemistry, Leibniz University of Hannover, Callinstraße 3, 30167 Hannover, Germany; 2 Laboratory of Biochemistry and Tumor Biology, Clinic of Obstetrics and Gynecology, Medical University, Hannover, Carl-Neuberg-Straße 1, 30625 Hannover, Germany

## Abstract

**Background:**

A variety of cell types can be identified in the adherent fraction of bone marrow mononuclear cells including more primitive and embryonic-like stem cells, mesenchymal stem cells (MSC), lineage-committed progenitors as well as mature cells such as osteoblasts and fibroblasts. Different methods are described for the isolation of single bone marrow stem cell subpopulations – beginning from ordinary size sieving, long term cultivation under specific conditions to FACS-based approaches. Besides bone marrow-derived subpopulations, also other tissues including human umbilical cord (UC) have been recently suggested to provide a potential source for MSC. Although of clinical importance, these UC-derived MSC populations remain to be characterized. It was thus the aim of the present study to identify possible subpopulations in cultures of MSC-like cells obtained from UC. We used counterflow centrifugal elutriation (CCE) as a novel strategy to successfully address this question.

**Results:**

UC-derived primary cells were separated by CCE and revealed differentially-sized populations in the fractions. Thus, a subpopulation with an average diameter of about 11 μm and a small flat cell body was compared to a large sized subpopulation of about 19 μm average diameter. Flow cytometric analysis revealed the expression of certain MSC stem cell markers including CD44, CD73, CD90 and CD105, respectively, although these markers were expressed at higher levels in the small-sized population. Moreover, this small-sized subpopulation exhibited a higher proliferative capacity as compared to the total UC-derived primary cultures and the large-sized cells and demonstrated a reduced amount of aging cells.

**Conclusion:**

Using the CCE technique, we were the first to demonstrate a subpopulation of small-sized UC-derived primary cells carrying MSC-like characteristics according to the presence of various mesenchymal stem cell markers. This is also supported by the high proliferative capacity of these MSC-like cells as compared to whole primary culture or other UC-derived subpopulations. The accumulation of a self-renewing MSC-like subpopulation by CCE with low expression levels of the aging marker senescence-associated β-galactosidase provides a valuable tool in the regenerative medicine and an alternative to bone-marrow-derived MSC.

## Background

MSC were first identified in the bone marrow [1] and characterized as a population of non-hematopoetic multipotent stem cells. Similar to other stem cell types MSC possess the potential for self-renewal and for differentiation into highly specialized cells upon appropriate stimulation. For example, MSC differentiation into cell types of the mesodermal lineage has been extensively investigated [2,3]. Moreover a variety of studies have demonstrated that MSC may also generate mature cells typically arisen from endoderm [4-6] or ectoderm [7-9] suggesting that cultures of bone marrow MSC may represent an admixture of phenotypically, functionally and biochemically different cells [10-12]. Indeed, besides MSC a variety of different cell types of predominantly mesodermal origin could be identified in the adherent fraction of bone marrow mononuclear cells including more primitive and embryonic-like stem cells, lineage-committed progenitors as well as mature cells such as osteoblasts and fibroblasts [13-16]. Therefore bone marrow MSC cultures appear to provide a broad spectrum of stem cells with various differentiation potential. However, the amount of primitive stem cells in these cultures is rare and can vary depending on the age of donor, method of cell isolation or cultivation respectively [17,18].

The research over the last decade has demonstrated that bone marrow is not the exclusive source for MSC. Cells with similar characteristics can be extracted from virtually all post-natal [19] as well as extra-embryonic tissues such as amniotic membrane [20], placenta [21] and UC [22-24]. However, the in vivo immunophenotype of MSC and distinct unique surface markers for the exact identification of MSC in the various tissues remains unclear [12]. In 2004, the International Society for Cellular Therapy appointed a set of standard criteria to facilitate a more uniform characterization of MSC. This current statement corroborates the prevalent opinion that the simultaneous expression of cell surface markers including CD44, CD73, CD90 and CD105 with a concomitant absence of CD45 and CD34 expression represents a specific phenotype for cultured MSC [25].

Different methods are described for the isolation of single bone marrow stem cell subpopulations – beginning from ordinary size sieving [26,27], long term cultivation under specific conditions [15,28,29] to FACS-based approaches [30,31] and previous work has suggested certain differentially-sized subpopulations of small, rapidly proliferating cells with high differentiation capacity [16,30]. In this context, it was the aim of the present study to identify possible subpopulations in cultures of MSC-like cells obtained from human UC and we are the first using CCE as a novel strategy to successfully address this question.

## Methods

### MSC-like cell isolation from umbilical cord tissue

Human umbilical cords were obtained from consenting patients (n = 3) delivering full-term (38–40 weeks) infants by Cesarean section. The use of this material has been approved by the Institutional Review Board, project #3037 in an extended permission on 17^th ^June, 2006. After removing the blood cells from the UC with PBS (phosphate buffered saline) enriched with 5 g/l glucose (Sigma Aldrich Chemie, Deisenhofen, Germany), 50 μg/ml gentamicin (PAA Laboratories GmbH, Pasching, Austria), 2.5 μg/ml amphotericin B (Sigma), 100 U/ml penicillin and 100 μg/ml streptomycin (PAA Laboratories GmbH), the UC tissue was cut into approx. 0.5 cm^3 ^large pieces and then incubated in αMEM (Invitrogen GmbH, Karlsruhe, Germany) reinforced with 15% of allogous human serum (kindly provided by the Division of Transfusion Medicine, Medical University Hannover, Germany) and 50 μg/ml gentamicin at 37°C in a humidified atmosphere with 5% CO_2_. The medium was changed every second day. A beginning outgrowth of an adherent cell layer from single tissue pieces was observed after approx. 10 days. After 2 weeks the UC tissue was removed and the adherent cells were harvested by accutase treatment according to the manufacturer's protocol (PAA Laboratories GmbH) for 5 min at 37°C. The obtained cell suspension was centrifuged at 200 × g for 5 min and the cells were resuspended in αMEM supplemented with 10% human serum and 50 μg/ml gentamicin and subcultured at a density of 4,000 cells/cm^2^. Following the second subconfluent passage, cells were harvested for the following characterization experiments or cryopreserved. Cryoconservation was performed with about 1.5 × 10^6 ^cells/ml in αMEM containing 10% (v/v) DMSO (Sigma) and 80% of human serum in liquid nitrogen.

### Counterflow Centrifugal Elutriation (CCE)

The CCE was performed using the Beckmann J6-MC with the JE-5.0 rotor and the appropriate 5 ml-standard elutriation chamber (Beckman Coulter GmbH, Krefeld, Germany) as previously described [32]. Approximately 4 × 10^8 ^MSC-like cells in an exponential growth phase were harvested, resuspended in PBS and applied to the standard chamber (1,600 rpm at 24°C) using a digital flow controller (Cole-Palmer Instruments Inc., Chicago, IL, USA). Subsequent fractions of 100 ml aliquots of the elutriated samples were collected upon progressive increase of the pump speed (table 1). Elutriated cell fractions were examined for viability, cell number and cell size distribution in a Vi-CELL Series Cell Viability Analyzer (Beckman).

**Table 1:** Parameters for CCE, cell size distribution and cell viability in the obtained CCE fractions and in the UC-derived primary cell population

**Fraction**	**Flow rate** **(ml/min)**	**Avg. diameter** **(μm)**	**Cell viability** **(%)**
1	0.8	11.1 ± 1.3	65.0 ± 15.3

2	1.2	12.4 ± 1.1	88.3 ± 0.7

3	1.,5	14.0 ± 1.9	94.5 ± 3.9

4	2.0	14.3 ± 1.0	86.9 ± 9.4

5	2.8	15.4 ± 1.1	80.7 ± 10.8

6 stop	2.8 (without centrifugation)	19.1 ± 3.1	75.1 ± 9.4

**Primary cell population **(control)	non-elutriated	15.0 ± 1.8	83.9 ± 5.2

### Phenotypic analysis by flow cytometry

MSC were harvested by use of accutase for 5 min at 37°C, recovered by centrifugation at 200 × g for 5 min, washed twice in ice-cold PBS supplemented with 2% FCS (PAA Laboratories GmbH) and resuspended to a concentration of about 10^5 ^cells/antibody test. Thus, 20 μL of a pre-diluted PE-conjugated mouse anti-human CD44, a PE-conjugated mouse anti-human CD73, a FITC-conjugated mouse anti-human CD90 antibody (all from BD Biosciences, Heidelberg, Germany) and a R-PE-conjugated mouse anti-human CD105 antibody (Invitrogen GmbH, Karlsruhe, Germany) was used, respectively. Negative control staining was performed using a FITC-conjugated mouse IgG1 κ isotype, a PE-conjugated mouse IgG1 kappa isotype (all BD Biosciences) and a R-PE-conjugated mouse IgG1 isotype antibody (Invitrogen), respectively.

After storage for 20 minutes at room temperature in the dark, 400 μL of PBS supplemented with 2% FCS were added and analyzed in the EPICS XL/MCL flow cytometer (Beckman Coulter GmbH). Living cells were gated in a dot plot of forward versus side scatter signals acquired on linear scale. At least, 10,000 gated events were acquired on a LOG fluorescence scale. Positive staining was defined as the emission of a fluorescence signal that exceeded levels obtained by >99% of cells from the control population stained with matched isotype antibodies. For the antigen expression which was normalized to cell size, the fluorescence of the conjugated monoclonal antibodies as well as the forward scatter signals were measured on linear scale. The ratios of fluorescence signals versus scatter signals were calculated by the EPICS XL/MCL flow cytometer (Beckman Coulter). Histograms were generated using the software WinMDI 2.8 (Joseph Trotter).

### Determination of cell proliferative activity

Immediately after CCE, the small-sized population (cells of the elutriation fraction 1), the large-sized population (cells of the elutriation fraction 6) and the UC-derived primary control population before elutriation was seeded at a density of 500 cells/cm^2 ^and cultivated in 25 cm^2 ^cell culture flasks (Sarstedt, Nuembrecht, Germany) in culture medium containing 10% human serum over 4 passages (P5–P8). After approx. 90% of confluency in the most rapidly proliferating subculture, cells from all three cultures were simultaneously harvested and replated at the same cell density. The cell number within the individual passages was determinate by the use of phase-contrast microscopy and trypan blue exclusion test.

### Determination of cell senescence

The amount of senescent cells was determined in the small-sized population (cells of the elutriation fraction 1), the large-sized population (cells of the elutriation fraction 6) and the UC-derived primary control population by the use of the Senescence β-Galactosidase Staining Kit (Cell Signaling Technology, Danvers, USA) and DAPI (4',6-Diamidin-2'-phenylindoldihydrochlorid) (Roche Diagnostics GmbH, Mannheim, Germany) fluorescence counterstain in accordance to the manufacturers' instructions. Thus, the 3 different populations were cultured for 6 days after elutriation, passaged and seeded at a density of 6,000 cells/cm^2 ^for 48 h before senescence-associated β-galactosidase (SA-β-gal) staining. After completion of the staining procedures, 4 representative images were taken from diverse areas of each cell culture using phase-contrast microscopy, fluorescence microscopy and Cell^B^Imaging Software (Olympus GmbH, Hamburg, Germany). For the calculation of the percentage of senescent cells the total number of cell nuclei and number of cell nuclei surrounded by cyan dye were enumerated.

## Results

### Cell fractions obtained during CCE

**Figure 1:**
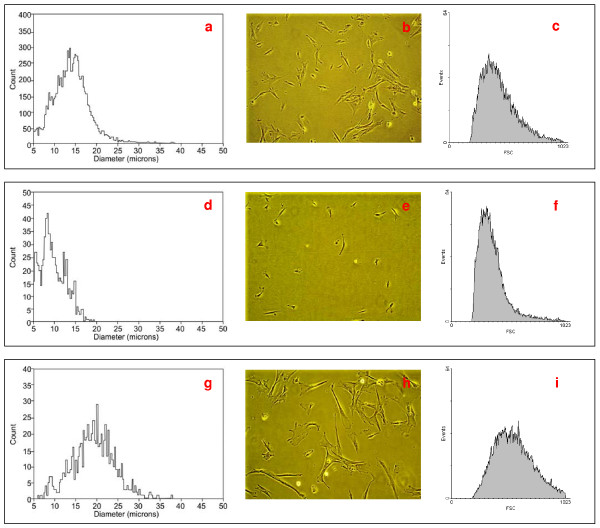
**Cell size distribution in the UC-derived primary culture (a, b, c) and in the subpopulations of small-sized (d, e, f) and large-sized (g, h, i) cells obtained after CCE**. Cell size was investigated using Vi-CELL Series Cell Viability Analyzer (a, d, g) and flow cytometry using FSC signals as a measure of cell size (c, f, i). Images of the corresponding cell cultures were taken on the next day after cell seeding using phase contrast microscopy (total magnification 100 ×) (b, e, h). Exemplary results of one representative experiment are presented (n = 3).

UC-derived primary cultures were found to be heterogeneous regarding cell size and morphology (figure 1a–c). Thus, large sometimes binucleated cells with a morphology varying from elongate to broad as well as small flat cells with an apparent increased nucleus-to-cytoplasm ratio were observed via phase-contrast microscopy (figure 1b). To further characterize this heterogeneous population, cell separation according cell size was performed using CCE.

During this procedure, six separate cell fractions with continuously increasing cell size were obtained (Table 1). Two clearly distinct cell subpopulations consisting of small cells with an average diameter of 11.1 ± 1.3 μm (elutriation fraction 1; figure 1d–f) and large cells with the diameter of 19.1 ± 3.1 μm (elutriation fraction 6; figure 1g–i) were isolated from primary cultures of human umbilical cord tissue. The small-sized subpopulation represented about 4,1% and the large-sized cells about 40% of the entire population. A similar distribution was observed in the UC-derived primary cultures of all 3 patients.

Cell size determination following elutriation was performed via Vi-CELL Series Cell Viability Analyzer (figure 1a, d, g) and revealed an initial viability of 65 ± 15% in the small cell population and 75 ± 9% in the large cell population (table 1). During subsequent culture the viability of these two populations increased to more than 90%, respectively. Analysis of the cultures by flow cytometry confirmed a different size distribution of the CCE-obtained subpopulations in the forward scatter (figure 1c, f, i). Moreover, cell cycle analysis demonstrated no significant differences in the cell cycle distribution of either the small-sized or the large-sized subpopulations with about 90% of cells in the G1/GO phase (data not shown).

### Characterization of the immunophenotype

**Figure 2:**
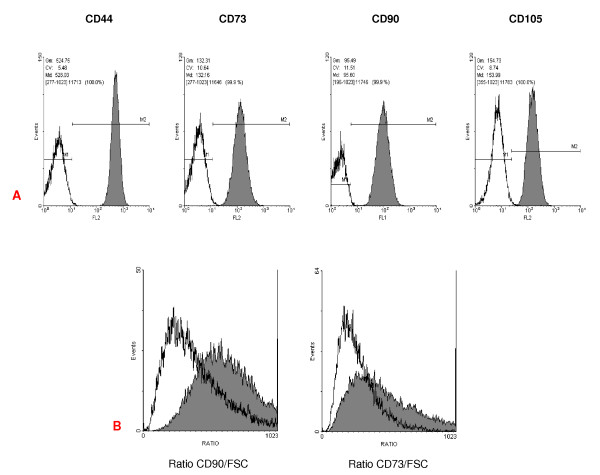
**Immunophenotype of cells obtained from human umbilical cord tissue**. **A**. Flow cytometric analysis of surface antigen expression in the UC-derived cultures was performed using the labelled antibody anti CD44-PE; anti CD73-PE; anti CD90-FITC; anti CD105-R-PE. At least 10,000 events are displayed. **B**. Exemplary CD90 and CD73 expression normalized on cell size: comparison of subpopulations of small- (filled histogram) and large-sized cells (unfilled histogram). The fluorescence of the conjugated monoclonal antibodies (anti CD90-FITC and anti CD73-PE) as well as the forward scatter (FSC) signals were measured on linear scale. The ratios were calculated by the EPICS XL/MCL flow cytometer (Beckman Coulter).

The immunophenotype of the UC-derived primary cultures was investigated via quantitative flow cytometry. All cells were highly positive for the surface antigens CD44, CD73, CD90 and CD105 (figure 2a). Moreover, expression of the surface molecules CD34 and CD45 were below detection limit (data not shown). A similar pattern of surface antigen expression was also observed in the differentially-sized subpopulations following CCE. However, quantitative flow cytometry when measured fluorescence signals of individual cells are divided by the corresponding forward scatter (FSC) signals revealed a decrease of antigen expression in the large-sized population (figure 2b, unfilled histograms) versus the small-sized cells (figure 2b, filled histograms) as demonstrated for CD73 and CD90, suggesting a more prominent expression of these mesenchymal stem cell markers in the small-sized population. The fluorescence distributions were compared using the Kolmogorov-Smirnov test and both populations were found to be different at the 99.9% confidence level.

### Proliferative activity of elutriated cells

**Figure 3:**
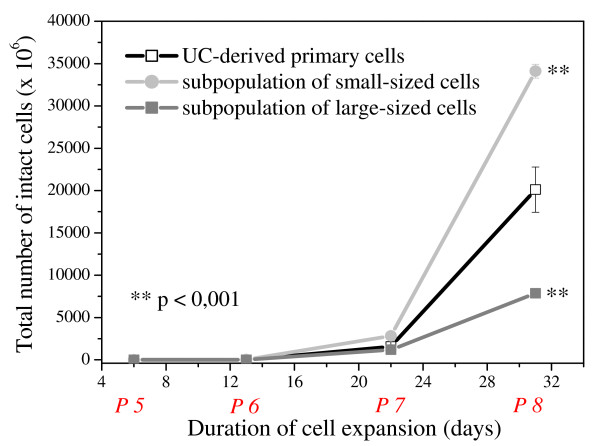
**Cell proliferative activity in cultures generated from the UC-derived primary cultures and from subpopulations of small- and large-sized cells**. Proliferation was measured by counting the total number of obtained intact cells. Following CCE, cells were seeded at a density of 500 cells/cm^2 ^and cultivated in αMEM containing 10% human serum over 4 passages (P5–P8) in quadruplicates. Student's t-tests were performed for the recognition of the significant differences (marked with asterisks) in comparison to UC-derived primary cell population.

Analysis of the proliferative capacity revealed the small-sized subpopulation most potent as compared to the original UC-derived primary cultures and the large-sized CCE-obtained subfraction. This increased proliferation potential in the small-sized subpopulation sustained during longer term culture at least until passage 8 after 31 days in culture (Fig. 3) Accordingly, the subpopulation of small cells passed 21.4 ± 0.03 cell population doublings after 31 days, whereas in the same time range the original UC-derived primary cultures reached 20.6 ± 0.3 and the subpopulation of large cells performed only 18.6 ± 0.2 cell population doublings. Exemplary data in quadruplicates of one representative experiment (n = 3) was demonstrated (Fig. 3).

### Analysis of senescence in the original UC-derived primary cultures and in the CCE-enriched small- and large-sized subpopulations

**Figure 4:**
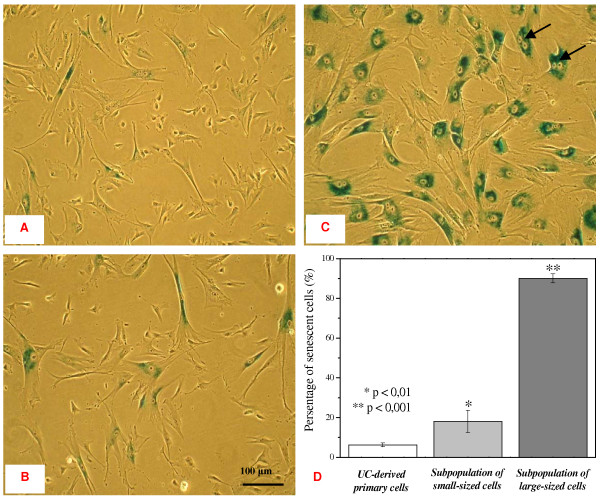
**SA-β-gal-positive cells in the UC-derived primary cell population (A) and in the CCE-derived subpopulations of small- (B) and large-sized (C) cells**. Cells were cultured for 6 days after elutriation. Following subculture, the cells were seeded at the density of 6,000 cells/cm^2 ^and cultured for further 48 h in complete medium. A relative high portion of binucleated cells (arrows) were detectable in the subpopulation of the large-sized cells. Student's t-tests were performed for the recognition of the significant differences (marked with asterisks) in comparison to UC-derived primary cell population.

Original UC-derived primary cultures and subpopulations of CCE-enriched small- and large-sized cells were cultivated over the same period for 6 days post elutriation. Following subsequent passage, the cells were seeded at a density of 6,000 cells/cm^2 ^and cultured for further 48 h. Exemplary images of the original primary culture (figure 4A), the small-sized subpopulation (figure 4B) and the large-sized cells (figure 4C) were obtained after SA-β-gal staining. Senescent cells are marked by cyan dye in the perinuclear area. Quantitative analysis revealed 6.3 ± 0.9% of senescent cells in the original primary culture and 18.0 ± 5.5% of senescence in the small-sized subpopulation. In contrast, 90.1 ± 2.3% of the large-sized subpopulation displayed features of a senescent phenotype (figure 4d). Thus, SA-β-gal positive and simultaneously binucleated cells were observed in the investigated cultures and appeared predominantly in the large-sized subpopulation indicating the presence of aberrant mitosis (figure 4C, arrows).

## Discussion

Culture of umbilical cord tissue pieces yielded an adherent growing cell population with a diverse morphology of small and large cells. Flow cytometric analysis revealed high levels of CD44, CD73, CD90 and CD105 expression whereas the expression of CD-proteins typical for hematopoietic cells remained undetectable. These findings suggested the presence of mesenchymal stem cell-like cells according to MSC standard criteria of the International Society for Cellular Therapy [25]. Moreover, a distinct subpopulation of the UC-derived primary cells demonstrated the potential to differentiate along the osteogenic pathway as evaluated by increase of alkaline phosphatase activity and increased mineralization (data not shown). This differentiation potential discriminated the UC-derived subpopulation from fibroblasts which are also most likely present in the original primary culture and carry a similar immunophenotype.

We observed marked morphological and cell size differences in the UC-derived primary cultures despite the rather homogeneous immunophenotype. For separation of these morphologically different cells, we applied CCE as a new approach. This technique yielded two subpopulations displaying distinct differences both, in cell size and morphology. Whereas the CCE-derived small-sized subpopulation exhibited the highest proliferative capacity and the most pronounced expression of mesenchymal stem cell markers, similar properties were observed in subpopulations of small-sized and rapidly-growing multipotential stem cells derived from human bone marrow [15,26,33].

Together, these findings suggested that the identified small-sized subpopulation of primary UC-derived cells exhibits MSC-like characteristics which may indicate a high relevance for applications in the area of regenerative medicine. This is also supported by the significant lower proliferative activity of the large-sized cells as well as the high portion of senescent cells in these cultures during long-term cultivation. In this context, the long-term expansion of the small-sized subpopulation was associated with a gradual loss of homogeneity displaying an enlarged and more diverse morphology. Accordingly, we speculate that the small cells may represent precursors of the larger, more mature cells, which eventually become senescent.

## Conclusion

CCE provides a useful approach to enrich small rapidly-proliferating MSC-like cells in mixed UC-derived primary cultures. However, further characterization of these subpopulations is required with respect to the differentiation capacity and the application potential in regenerative medicine.

## Abbreviations

MSC: (mesenchymal stem cells); UC: (umbilical cord); CCE: (counterflow centrifugal elutriation); FSC: (forward scatter)

## Competing interests

The authors declare that they have no competing interests.

## Authors' contributions

IM conceived and participated in the design of the study, carried out the MSC-like cell isolation and expansion, cell proliferation and senescence studies, performed statistical analysis, analysis and interpretation of data, as well as drafted the manuscript. PM carried out the determination of cell size and immunophenotype via flow cytometry, performed statistical analysis as well as analysis and interpretation of received flow cytometry data, helped to draft the manuscript. RH participated in the design of the study, carried out counterflow centrifugal elutriation, cell size determination via Vi-CELL Series Cell Viability Analyzer and performed a critical revision of the manuscript. CK participated in the design and coordination of the study. All authors read and approved the final manuscript.
